# Realization of Quantum Secure Direct Communication with Continuous Variable

**DOI:** 10.34133/research.0193

**Published:** 2023-07-14

**Authors:** Zhengwen Cao, Yuan Lu, Geng Chai, Hao Yu, Kexin Liang, Lei Wang

**Affiliations:** School of Information Science and Technology, Northwest University, Xi’an710127, China.

## Abstract

With the progress of theoretical and applied technologies, the communication system based on the classical encryption is seriously threatened by quantum computing and distributed computing. A communication method that directly loads confidential information on the quantum state, quantum secure direct communication (QSDC), came into being for resisting security threats. Here, we report the first continuous-variable QSDC (CV-QSDC) experimental demonstration for verifying the feasibility and effectiveness of the CV-QSDC protocol based on Gaussian mapping and propose a parameter estimation for signal classification under the actual channels. In our experiment, we provided 4 × 10^2^ blocks, where each block contains 10^5^ data for direct information transmission. For the transmission distance of 5 km in our experiment, the excess noise is 0.0035 SNU, where SNU represents the unit of shot-noise units. The 4.08 × 10^5^ bit per second experimental results firmly demonstrated the feasibility of CV-QSDC under the fiber channel. The proposed grading judgment method based on parameter estimation provides a practical and available message processing scheme for CV-QSDC in a practical fiber channel and lays the groundwork for the grading reconciliation.

## Introduction

In an era of highly developed network technology, there are still some insecure factors in the transmission of confidential information. One-time pad is a mechanism of communication that theoretically guarantees the absolute security of encrypted information [1]. Its security is guaranteed by the security of the key. However, with the progress of theoretical and applied technologies, the communication system based on the classical encryption is seriously threatened by quantum computing and distributed computing [2–4].

In order to resist security threats that are caused by brute-force cracking of key by increasing computational complexity, a way of communication based on fundamental principles of quantum mechanics came into being. Different from quantum key distribution (QKD) [5–8], quantum secure direct communication (QSDC) is the true sense of secure communication, and its secret message is loaded on the quantum state and transmitted directly in the channel [9]. Quantum principles such as no-cloning theorem [10], Heisenberg’s uncertainty principle [11], and the correlation of entangled particles [12] guarantee its security. Moreover, this mode retains the function of perceiving eavesdropping while making it impossible for Eve to eavesdrop any secret message, that is, to achieve the role of anti-eavesdropping. Therefore, the development of this technology is conducive to the conversion of classical communication to quantum communication.

Generally, discrete-variable (DV) scheme and continuous-variable (CV) scheme are 2 important technologies to realize quantum communication. Since the first DV protocol was proposed, DV-QSDC was well developed in recent years in both theory and experiment [13–18]. For instance, device-independent (DI) QSDC and measurement device-independent (MDI) QSDC protocols have been proposed [19–27]. Additionally, one-step QSDC protocols and their DI and MDI versions, which enable the transmission of photons in a single round, have also been introduced [28–30]. Focusing on the experimental demonstration, in the original scheme proposed by Long and colleagues [31,32], optical delay, a mature and experimentally feasible technique that can be used to replace quantum memory, makes full use of the 2-qubit in an Einstein-Podolsky-Rosen (EPR) pair to implement 2-step DV-QSDC experiments. On this basis, many experimental schemes have been further proposed. In 2016, Long and colleagues [33] reported the first experimental demonstration of DV-QSDC based on the DL04 protocol, and the feasibility was firmly demonstrated in the presence of noise and loss. Zhang et al. [34] first reported the experimental demonstration with atomic quantum memory for the first time in principle in 2017, demonstrating the potential application of long-distance quantum communication in quantum networks. In 2018, Sun et al. [35] proposed an implementation scheme without quantum memory, taking advantage of the wiretap channel theory and classical coding theory, and made great progress in the practical application of QSDC. In 2019, Long and colleagues [36] implemented a practical DV-QSDC system based on the DL04 protocol that operates at a repetition rate of 1 MHz under a realistic channel of 1.5 km with high noise and high loss characteristics. The secure communication rate achieves 50 bps, sufficient to effectively send text messages and reasonably sized files of images and sounds. In 2020, Sun et al. [37] proposed a quantum-memory-free DL04 QSDC protocol inches closer to the quantum channel’s capacity and importantly improved the original DL04 QSDC’s robustness. Pan et al. [38], also in 2020, reported an experimental implementation of free-space DV-QSDC, which shows that QSDC is feasible in free space. In 2021, Liu et al. [39] designed a 2-decoy-state protocol that only requires 2 decoy states and performed full parameter optimization for a real-life QSDC system by introducing a genetic algorithm. In 2022, Liu et al. [40] presented a fiber-based QSDC system without active polarization compensation. Long and colleagues [41] successfully experimentally implemented DV-QSDC of over 100 km fiber with time-bin and phase quantum states also in 2022, demonstrating that intercity QSDC through fiber is feasible with present-day technology.

Compared with DV scheme, CV quantum communication can be well compatible with the existing optical fiber communication, which has been extensively studied recently. Moreover, its advantages of simple light source preparation, convenient detection, and low equipment cost are conducive to the construction of quantum communication network. Therefore, in order to accelerate the development of the entire QSDC field, it is necessary to explore CV-QSDC technology.

The principles of quantum mechanics impose an upper bound on the information in CV-QSDC that has possibly leaked to a potential eavesdropper, which is done by the legitimate parties through estimating the parameters of quantum channels [42–44]. In the preliminary development of QSDC, parameter estimation, a crucial aspect connecting quantum information processing stage and message extracting stage, has attracted the attention of many researchers. In addition, parameter estimation can also be combined with other methods that suppress excess noise, such as adaptive optics [45], to improve system performance. However, numerous schemes that have been proposed so far are only for CVQKD [46–51]. Different from QKD, QSDC that directly transmits information has more elaborate requirements for parameter estimation, where the complete extraction of subsequent information cannot be effectively guaranteed by those existing schemes. Therefore, it is necessary to research on parameter estimation scheme suitable for system based on experiments.

In this context, we established an experimental CV-QSDC platform based on Gaussian mapping scheme [52] and experimentally demonstrated it. We introduced a new parameter estimation scheme under the actual channels with complexity and fluctuation. Specifically, the main contributions of this work are as follows: (a) We reported the first CV-QSDC experimental demonstration for verifying the feasibility and effectiveness of the protocol based on Gaussian mapping. Experimental results show that the proposed scheme successfully implements under the actual channel that is complex and volatile. (b) We performed the parameter estimation that came closer to the actual situation through the experimental demonstration and simulated the influence of whether the channel fluctuation is taken into account on the transmission distance. The results obtained from parameter estimation also more realistically and accurately reflect the fluctuation and instability of the actual channel, which may have a great impact on the efficiency of subsequent message extraction. Moreover, it can be obtained experimentally that the system performance can be improved if the channel fluctuation characteristics are taken into account. (c) We proposed a scheme for quality classification of information blocks based on parameter estimation results, which further optimizes the process of sequential postprocessing to improve the security and integrity of message. Furthermore, this scheme facilitates the extraction of secret messages from continuous variables and lays the technical foundation for the complete construction of the system and future engineering applications.

## Results

### System implementation

**Fig. 1.**
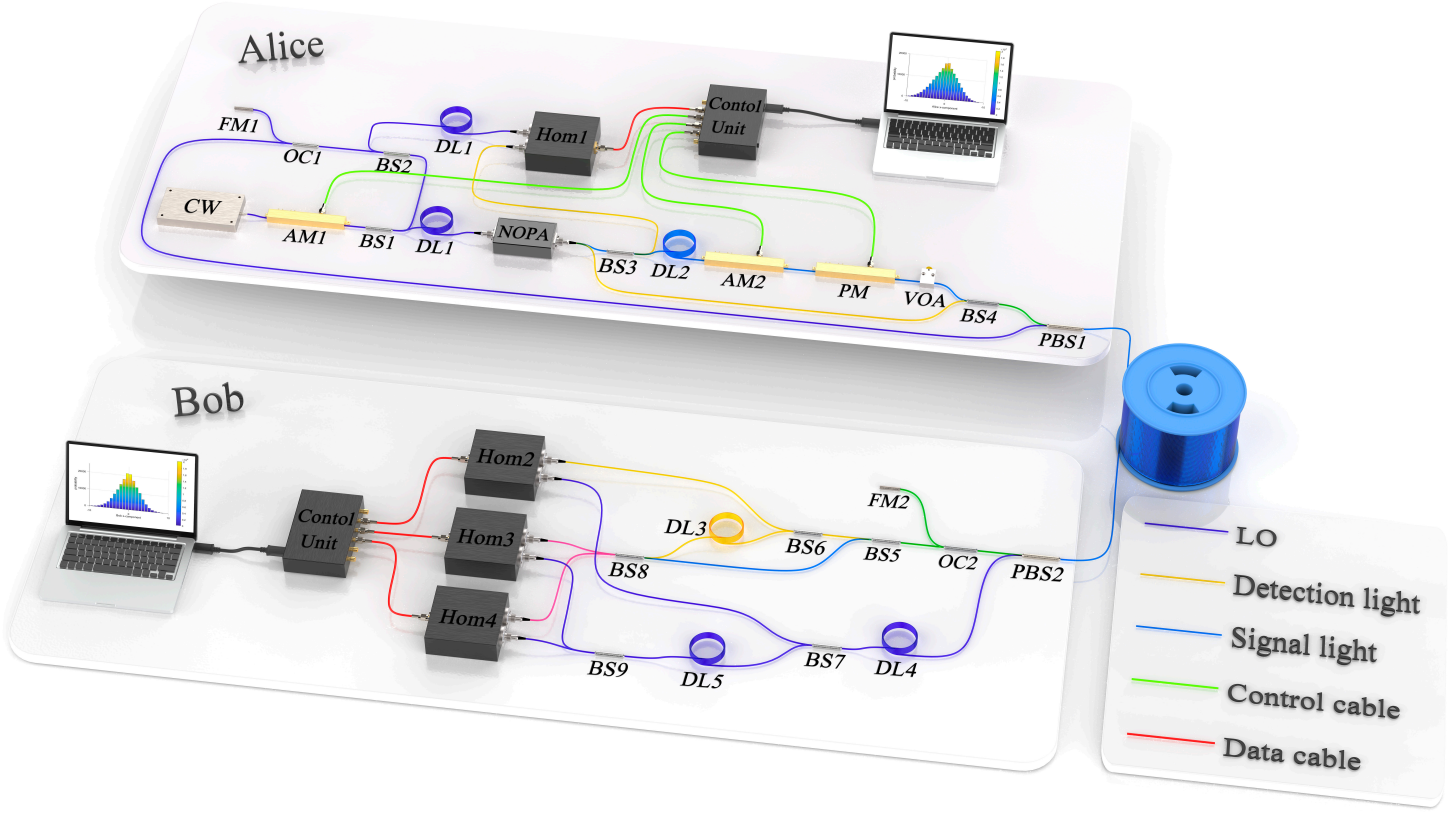
Experiment setup. CW, 1550-nm continuous-wave light source; NOPA, nondegenerate optical parametric amplifier; BS, beam splitter; OC, optical circulator; DL, fiber delay line; AM, amplitude modulator; PM, phase modulator; VOA, variable optical attenuator; Hom, homodyne detector with over 50% detection efficiency; PBS, polarization beam splitter; FM, faraday mirror.

The experimental setup for the proposed CV-QSDC system is shown in Fig. 1. The necessary conditions for CV-QSDC experiment include the coherent light sources (CW) for generating entangled light, the modulators (AM2 and PM) for information modulation, the homodyne detectors (Hom3 and Hom4) for Bell detection, and pilot signal for synchronization. Utilization of polarization multiplexing and time-division multiplexing ensures the transmission of local oscillator light, detection light, and signal light without mutual interference. Concretely, to verify the feasibility of the proposed CV-QSDC protocol based on Gaussian mapping scheme, coherent light source CW for generating coherent light, nondegenerate optical parametric amplifier NOPA, and amplitude modulator AM1 are used in experiments to generate entangled light pulses at a repetition rate of 10 MHz, where the resulting signal light is retained at Alice for information modulation and the detection light is sent to Bob for channel secure detection. The beam splitter BS3 and homodyne detector Hom1 at Alice’s end and BS6 and Hom2 at Bob’s end are adopted for channel secure detection. Meanwhile, signal light passes through the fiber delay line DL2 and then completes the modulation of the secret message under the joint action of the amplitude modulator AM2 and the phase modulator PM, and then passes through the polarization beam splitter PBS1 and fiber channel to Bob.

**Fig. 2.**
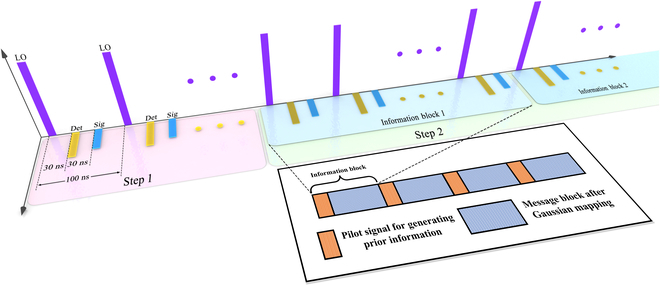
Schematic diagram of signal transmission during communication. Step 1 represents the security detection phase, while step 2 represents the information transmission phase. Specifically, polarization multiplexing and time-division multiplexing are employed to differentiate the local oscillator (LO), detection light (Det), and signal light (Sig), and the time interval for time-division multiplexing is set to 30 ns. During the information transmission phase, each information block consists of pilot signal and secret message. The pilot signal consists of quantum light [54] and undergoes the same channel as the transmitted signal light. Therefore, the a priori information obtained from these pilot signals and the *X* component can be used for synchronization and calculating the secrecy capacity.

The entire communication process consists of 2 steps. The security detection is conducted first, and information transmission occurs only under the premise of the security of channel. As shown in Fig. 2, the local oscillator, the detection light, and the signal light are transmitted using polarization multiplexing and time-division multiplexing techniques through Faraday mirror and fiber delay lines. Specifically, during the secure detection phase, Alice and Bob perform measurements on their respective entangled light beams in the selected corresponding time slots. So, here, taking fiber delay line into account,

$$\Delta t=\frac{n\Delta L}{c},$$ (1)

where Δ*L* is the length of fiber delay line, *n* is the refractive index of optical fiber, and *c* is the speed of light in vacuum [53]. Due to the repetition frequency of 10 MHz, the interval between identical pulses is 100 ns. Therefore, the 30-ns fiber delay is used in the experiment (shown in Fig. 2).

When Bob receives the modulated signal light, depolarization demultiplexing and time-division demultiplexing are performed to separate the detection light and the signal light for performing Bell measurements (employing BS8, Hom3, and Hom4). The quantum memory is not required in this experimental platform, mainly because the CV-QSDC protocol based on Gaussian mapping is actually a secure coding protocol based on Wyner’s wiretap channel theory. The plaintext to be transmitted is mapped into ciphertext in groups using shared Gaussian random sequence, and generated ciphertext is then transmitted through a quantum channel using error correction codes and detection codes.

**Fig. 3.**
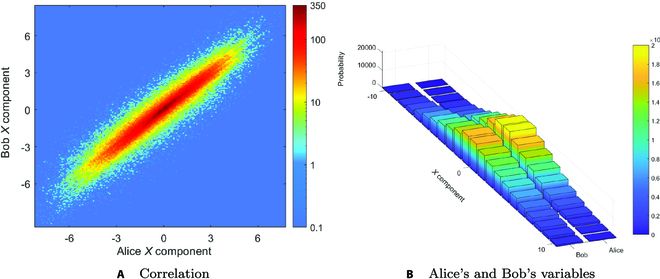
Experimental data renderings. (A) Correlation between Alice and Bob variables. The solid red line indicates that the data satisfy the linear relationship of *y* = *tx* + *z*. (B) Statistical properties of the *X* component of Alice and Bob. The horizontal axis represents the variables, while the vertical axis represents the frequency.

The complete CV-QSDC system functions mainly include the entanglement judgment function during the first safety inspection and the information transfer function based on entangled state. The experimental results verified the correlation of data in the CV-QSDC protocol based on Gaussian mapping under 5-km channel and effectiveness of the parameter estimation. The rendering of partial variables acquired from the quadrature component *X* obtained experimentally is shown in Fig. 3. In particular, correlation of variables between Alice and Bob is shown in Fig. 3A. The experimental results prove that most of the variable points are linear correlation, which verifies the feasibility of the implementation for the proposed scheme. The statistical characteristics of Alice and Bob variables are shown in Fig. 3B. The experimental results show that the variables at both ends are in line with the Gaussian distribution, indicating that the experimental variables can meet the necessary conditions for carrying out subsequent steps. Therefore, the above experiment shows the rationality of variable acquisition and provides data support for the message extracting stage.

### Parameter estimation scheme

The secret message on the basis of obtaining quadrature components is extracted by Alice and Bob during the message extracting stage. The processing operations mainly include the following 3 stages: parameter estimation, data reconciliation, and message extraction. Among them, parameter estimation plays a critical role in connecting the quantum information processing and message extracting in the CV quantum communication system. The results of parameter estimation can provide a reference for achieving the consistency of the data of both parties. Most studies in the past have assumed that the relevant parameters are theoretically invariant when analyzing the system performance [42,55–58], but this assumption is not in line with the actual situation. Next, a appropriate parameter estimation scheme is described.

In the CV-QSDC system, the message is transmitted in chunks rather than in whole. For each information block, secrecy capacity Δ*I_i_* of the *i*-th information block is calculated based on the estimated value of the parameters. After the measure by Bob, Alice and Bob possess 2 sets of related variables, namely, $X={\left\{\left(x_{i}^{A},x_{i}^{B}\right)\right\}}_{i=1,2,\ldots ,M}$ and $P={\left\{\left(p_{i}^{A},p_{i}^{B}\right)\right\}}_{i=1,2,\ldots ,M}$ (superscripts *A* and *B* represent the variables obtained by Alice and Bob, respectively, subscript *i represents the i-th information block, M* represents the total number of information block). One set of variables carries secret message, and the other set of variables carries Gaussian random numbers without secret message, which is the set of data samples of parameter estimation. It is assumed here that the secret message is modulated on the *P* component, while the *X* component is used for parameter estimation. Generally speaking, all the blocks on the *X* component are employed to estimate the relevant channel parameters. Among them, the transmittance *T* and excess noise *ε* of quantum channel can be used to describe the influence of the transmission on the quantum signal [59].

The quantum channel is described through linear models [60] in actual phenomenon. So, here, taking the correlation of variables of the *i*-th information block between Alice and Bob into account,

$$x_{i}^{B}=x_{i}^{A}t_{i}+z_{i},$$ (2)

where $t_{i}=\sqrt{\eta T_{i}}$, and *z_i_* is Gaussian noise with variance $\sigma_{i}^{2}=\eta T_{i}\varepsilon_{i}+N_{0i}+v_{\mathrm{el}}$ and mean zero, and *N*_0*i*_ is the shot noise. *T_i_* and *ε_i_* are the transmittance and excess noise of the *i*-th information block, respectively. Detecting efficiency *η* and the electrical noise *v_el_* induced by the detector can be obtained by precalibration. The variance and quantum communication system parameters meet the following relationships:

$$\begin{array}{ll} \langle {x_{i}^{A}}^{2}\rangle & =V_{A},\;\langle x_{i}^{A}x_{i}^{B}\rangle =\sqrt{\eta T_{i}}V_{A},\\ \langle {x_{i}^{B}}^{2}\rangle & =\eta T_{i}V_{A}+N_{0i}+\eta T_{i}\varepsilon_{i}+v_{\mathrm{el}}, \end{array}$$ (3)

where *T*_i_, *V_A_*, *N_0i_*, *ε_i_*, and *v_el_* all expressed in their respective units rather than in shot noise units. Since detecting efficiency *η* and electrical noise *v_el_* are calibrated in advance, Bob can obtain ${\left\{{}_{j}x_{0}^{B}\right\}}_{j=1,2,\ldots ,N^{\prime }}$ (*N'* represents the total number of quantum signals required to transmit an information block over quantum channel) by forcing a quantum channel not to transmit any signals. So the set of Bob’s variables ${\left\{{}_{j}x_{0}^{B}\right\}}_{j=1,2,\ldots ,N^{\prime }}$ can be employed to calibrate the noise of the *i*-th information block,

$$\langle {\left({}_{j}x_{0i}^{B}\right)}^{2}\rangle =N_{0i}+v_{\mathrm{el}}.$$ (4)

So the maximum likelihood estimate for the corresponding parameters based on Eq. 2 and the variables possessed by both communication parties can be obtained:

$$\begin{array}{ll} {\hat{\sigma }}_{0i}^{2}\; & =\frac{1}{N^{\prime }}\sum_{j=1}^{N^{\prime }}‍{\left({}_{j}x_{0i}^{B}\right)}^{2},\;{\hat{V}}_{A}=\frac{1}{N}\sum_{j=1}^{N}‍{\left({}_{j}x_{0i}^{A}\right)}^{2},\\ {\hat{t}}_{i}\; & =\frac{\sum_{j=1}^{N}{‍}_{j}{x_{i}^{A}}_{j}x_{i}^{B}}{\sum_{j=1}^{N}‍{\left({}_{j}x_{i}^{A}\right)}^{2}},\;{\hat{\sigma }}_{i}^{2}=\frac{1}{N}\sum_{j=1}^{N}‍{\left({}_{j}x_{i}^{B}{-}_{j}x_{i}^{A}\hat{t}\right)}^{2}, \end{array}$$ (5)

where *N* represents the total number of quantum signals required to transmit in each information block, ${\hat{\sigma }}_{0}^{2}$, ${\hat{V}}_{A}$, ${\hat{t}}_{i}$, and ${{\hat{\sigma }}_{i}}^{2}$ are independent estimates of each other and are subordinated to the following distributions:

$$\begin{array}{cc} & {\hat{t}}_{i}∼N\left(t_{i},\frac{\sigma_{i}^{2}}{\sum_{j=1}^{N}‍{\left({}_{j}x_{i}^{A}\right)}^{2}}\right),\\ & \frac{N{\hat{\sigma }}_{i}^{2}}{\sigma_{i}^{2}},\frac{N^{\prime }{\hat{\sigma }}_{0i}^{2}}{\sigma_{0i}^{2}},\frac{N{\hat{V}}_{A}}{V_{A}}∼\chi^{2}\left(N-1\right), \end{array}$$ (6)

where $\sigma_{0}^{2}$, *V*_A_, *t_i_*, and $\sigma_{i}^{2}$ are the truth values of the corresponding parameters [42,61]. The estimation following the *χ*^2^ distribution can be approximated by the Gaussian distribution when the sample size is sufficient, and the corresponding confidence interval can be expressed as:

$$\begin{array}{ll} t_{i}\; & \in \left[{\hat{t}}_{i}-\Delta t_{i},{\hat{t}}_{i}+\Delta t_{i}\right],\\ \sigma_{i}^{2}\; & \in \left[{\hat{\sigma }}_{i}^{2},-\Delta \sigma_{i}^{2},{\hat{\sigma }}_{i}^{2}+\Delta \sigma_{i}^{2}\right],\\ V_{A}\; & \in \left[{\hat{V}}_{A}-\Delta V_{A},{\hat{V}}_{A}+\Delta V_{A}\right],\\ \sigma_{0}^{2}\; & \in \left[{\hat{\sigma }}_{0}^{2},-\Delta \sigma_{0}^{2},{\hat{\sigma }}_{0}^{2}+\Delta \sigma_{0}^{2}\right], \end{array}$$ (7)

where Δ*t_i_*, $\Delta \sigma_{i}^{2}$, Δ*V*_A_, and $\Delta \sigma_{0}^{2}$ are given by

$$\begin{array}{ll} \Delta t_{i} & =z_{\varepsilon_{\mathrm{PE}}/2}\sqrt{\frac{{\hat{\sigma }}_{i}^{2}}{NV_{A}}},\;\Delta \sigma_{i}^{2}=z_{\varepsilon_{\mathrm{PE}}/2}\frac{{\hat{\sigma }}_{i}^{2}\sqrt{2}}{\sqrt{N}},\\ \Delta V_{A} & =z_{\varepsilon_{\mathrm{PE}}/2}\frac{{\hat{V}}_{A}^{2}\sqrt{2}}{\sqrt{N}},\;\Delta \sigma_{0}^{2}=z_{\varepsilon_{\mathrm{PE}}/2}\frac{{\hat{\sigma }}_{0}^{2}\sqrt{2}}{\sqrt{N^{\prime }}}. \end{array}$$ (8)

In addition, *z*_*ε*_PE_/2_ satisfies the equations:

$$\left\{\begin{array}{cc} & 1-\text{erf}\left(\frac{Z_{\varepsilon_{\mathrm{PE}}/2}}{\sqrt{2}}\right)=\frac{\varepsilon_{\mathrm{PE}}}{2},\\ & \text{erf}\left(x\right)=\frac{2}{\sqrt{\pi }}\int_{0}^{x}‍\text{exp}\left(-\tau^{2}\right)d\tau , \end{array}\right.$$ (9)

where *ε*_PE_ is the probability that the parameter estimate is outside the confidence interval calculated during the parameter estimation phase. Finally, the estimated values of parameters can be obtained:

$${\hat{T}}_{i}=\frac{{\hat{t}}_{i}^{2}}{\eta },\;{\hat{\varepsilon }}_{i}=\frac{{\hat{\sigma }}_{i}^{2}-{\hat{\sigma }}_{0i}^{2}}{{\hat{t}}_{i}^{2}}.$$ (10)

In the process of parameter estimation, the estimation of modulation variance ${\hat{V}}_{A}$, transmittance $\hat{T}$, and excess noise $\hat{\varepsilon }$ has the most obvious effect on the calculation of the secrecy capacity. However, the modulation variance of an information block is a set fixed value, estimation of which is close to the original modulation variance. Therefore, the effect on the estimation of secrecy capacity is mainly determined by the transmittance and excess noise in the channel.

To perform the parameter estimation, the experiment sets the total information to 4 × 10^2^ blocks, and each block contains 10^5^ variables. The overview of experimental parameters and performance is shown in Table.

**Table.** Overview of experimental parameters and performance. *SNR*, signal-to-noise ratio; *β*, reconciliation efficiency; *FER*, frame error rate of the reconciliation; *V_A_*, modulation variance; *ξ*, excess noise at the channel input; *v_el_*, electronic noise; *η*, efficiency of the homodyne detector; *P*, error rate of information block; *SC*, secrecy capacity; bps, bit per second.

Parameter	*Length *(km)	*SNR *(dB)	*β*	*FER*	*V_A_* (SNU)
Value	5	1.895	0.89	10^−3^	7
Parameter	*ξ* (SNU)	*v_el_* (SNU)	*η* (SNU)	*P*	*SC* (bps)
Value	0.0035	0.0042	0.6	10^−5^	4.08 × 10^5^

**Fig. 4.**
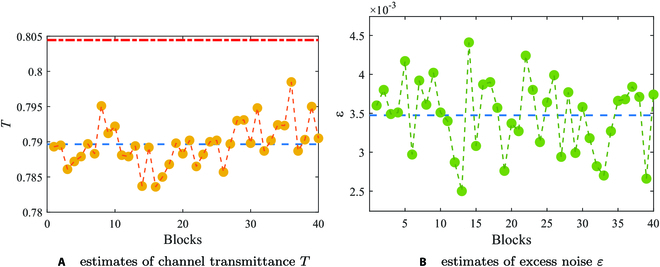
(A and B) Parameter estimates of channel transmittance and excess noise. The small solid circles in subgraphs represent the result of estimation of 40 information blocks selected as observation objects. The blue dotted line represents the average of the parameter estimates of all information blocks. The volatility of the data is represented by the dotted lines connecting points to points. The red dotted line in (A) represents the theoretical upper bound of channel transmittance.The optical fiber model used in our experimental platform is G652D, with an actual attenuation coefficient *α* of approximately 0.19 dB/km. Therefore, the theoretically calculated upper limit of the channel transmittance is approximately 0.804.

The result estimates of channel transmittance and excess noise are shown in Fig. 4. According to the distribution of small solid circles in Figs. 4A and B, the experimental results demonstrate the feasibility of the existing parameter estimation scheme in estimating the channel transmittance and excess noise, and lay the foundation for the design and implementation of subsequent grading scheme. In addition, the volatility nature of the data is obviously shown. Low-quality information blocks are more severely affected by channel interference and eavesdropping interference, making it more challenging to extract secure messages from them compared to high-quality information blocks. When using the same reconciliation scheme, the reconciliation efficiency of low-quality information blocks is lower than that of high-quality information blocks. Therefore, the grading processing based on different quality information blocks is highly necessary. By employing a more efficient reconciliation scheme for low-quality information blocks, the aim is to enhance their reconciliation efficiency and ultimately achieve high reconciliation efficiency for different quality information blocks.

**Fig. 5.**
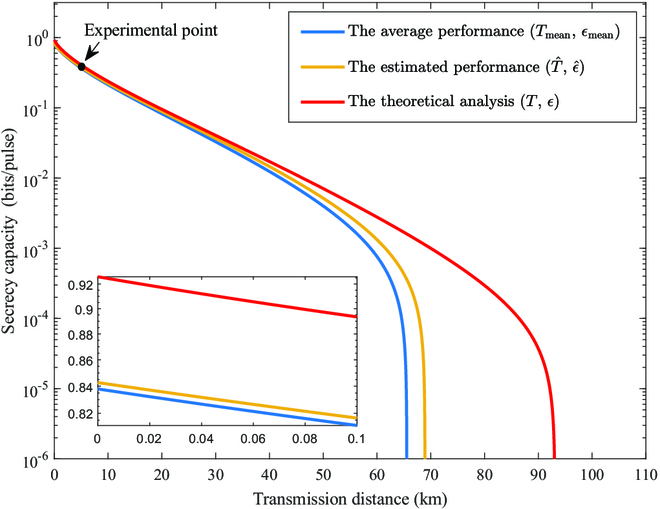
The relationship between the transmission distance and the secrecy capacity, which is based on the calibrated parameters in Table 1 using a standard expression. From left to right: Average performance result of the system, performance of a system that takes into account channel volatility, and theoretical performance of the system.

The relationship between the transmission distance and the secrecy capacity under different conditions is shown in Fig. 5. The transmission performance of the result based on the parameter estimate under conditions that take channel volatility into account is represented by the second curve from left to right. For a transmission distance of 5 km in our experiment, the excess noise is 0.0035 SNU, where SNU represents the unit of shot-noise units. The secrecy capacity has reached 4.08 × 10^5^ bit per second (bps), which demonstrates the effectiveness of the experimental platform. Obviously, compared to the average performance (the blue curve), the safe transmission distance is increased and the system performance is improved when parameter estimation under conditions that take volatility into account (the yellow curve).

**Fig. 6.**
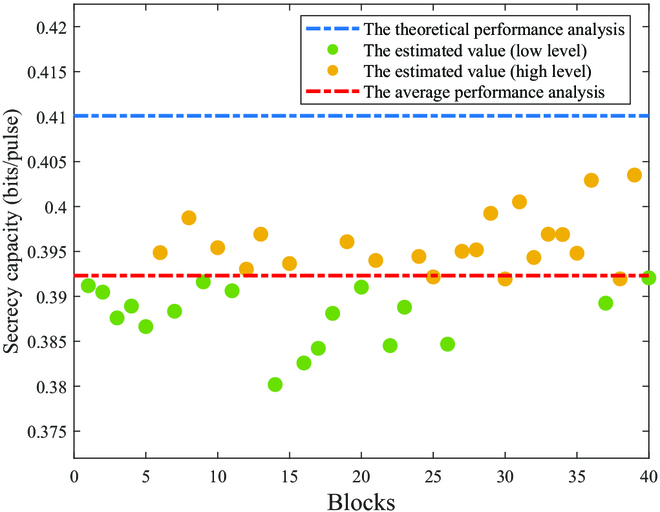
The estimate of secrecy capacity. The data points represent 40 information blocks selected as grading objects. The blue dotted line indicates the secrecy capacity in theoretical case when the transmission length of the fiber channel is set to 5 km. The red dotted line indicates the average of secrecy capacity, which is obtained by calculating the secrecy capacity for multiple segments of pilot signals and then taking the mean. The information blocks that satisfy criterion 2 are represented by the yellow data point. The information blocks that do not meet criterion 2 are represented by the green data point.

Based on the above analysis, a grading processing operation explained in detail in the “Grading processing method based on parameter estimation” section is adopted to classify the quality of the information blocks, so as to perform the corresponding reconciliation strategies on the information blocks of different quality to improve the reconciliation efficiency of information blocks and speed up the extraction rate of secret message. The calculation results of grading all information blocks are shown in Fig. 6, which shows that the experimental communication process is secure and explains that the proposed grading processing scheme is feasible and effective.

## Discussion

In this work, an experimental platform is established for the Gaussian mapping-based CV-QSDC. The experiment completes the process of CV-QSDC on the platform basis well, and the experimental data from both parties have a strong correlation. According to Fig. 6, the curve fitted from the data suggests that the secrecy capacity of this system can reach 10 bps at 100 km. Compared to the currently achievable DV-QSDC experimental system with the longest communication distance in the world, this experimental system has a much higher communication rate than its rate of 0.54 bps at 100 km [41]. This comparison highlights the advantage of the established experimental platform in terms of communication rate. However, one limitation of the continuous-variable experimental system is the high signal loss and limited transmission distance in the channel, making it suitable only for short- to medium-distance communication. Therefore, the future development goal for continuous-variable experimental system is to enhance the communication distance as much as possible. The results of the experiment also show that the platform has the advantages of systematization, extensiveness, and comprehensiveness and has certain reference value and guidance significance to the study of parameter estimation and secret message grading processing part of CV-QSDC. With the help of the proposed parameter estimation scheme and the analysis of beam propagation in the fiber channel based on the established platform, the transmittance and the excess noise of system have been studied for in-depth analysis of the actual performance of the system. The experimental results show that the proposed parameter estimation scheme has effectiveness in estimating the channel transmittance and excess noise. The estimation of secrecy capacity and grading criteria of information in this protocol are also effective. Moreover, the experimental results show the rationality of data acquisition and provide data support for the message extracting stage.

Consequently, the experimental result based on this experimental platform coincides with the relevance characteristics of the message before and after the transmission for CV-QSDC, which proves the availability and rationality of this platform and provides a feasible method for experiment of CV-QSDC. Furthermore, by incorporating coding processes, employing transmission, and receiving antennas at the communication endpoints, this system has the potential for future development in CV-QSDC communication in free space. Moreover, the experimental result based on the parameter estimation including grading judgment provides a practical and available post-processing method for CV-QSDC in a practical fiber channel. Finally, based on the analysis mentioned above, the established platform can acquire data appropriately and efficiently provide data support for the message extracting stage. The proposed parameter estimation method can effectively improve the grading processing performance and lay the groundwork for the grading processing reconciliation scheme. All in all, future work will therefore focus on solving the problem of adaptation between information grading reconciliation schemes and different transmission quality information blocks under actual experimental systems.

## Materials and Methods

### The CV-QSDC protocol based on Gaussian mapping

The CV-QSDC protocol based on Gaussian mapping, employing Gaussian source and Gaussian modulation, is proposed to solve the offset problem present in existing CV-QSDC protocol [62]. Specifically, the Gaussian random number sequence, directly encoded by the transmitted secret message, has nonuniform characteristics, which lead to the difference of the mean in the subsequent modulation process. The instability of the mean affects the modulation variance, which is crucial to the quantum communication process. This further impacts the message extraction, resulting in the compromise of the actual security of the system. Through Gaussian mapping, the information in each block is uniformized, which ensures the stability of Gaussian modulation under different variance conditions and facilitates the secure message transmission.

•**Protocol structure**

1. **Preparation**: Optical parameter oscillator is employed to compress the quadrature position (*x*) or quadrature momentum (*p*) of the vacuum state in phase space to generate 2 squeezed vacuum light (beam 1 and beam 2). The quadrature position and quadrature momentum corresponding to the squeezed vacuum states can be expressed as:

$$\left\{\begin{array}{cc} x_{1}\; & =\text{exp}\left(-r\right)|0\rangle {}_{x_{1}},\\ p_{1}\; & =\text{exp}\left(r\right)|0\rangle {}_{p_{1}}, \end{array}\right.$$ (11)

and

$$\left\{\begin{array}{cc} x_{2} & =\text{exp}\left(-r\right)|0\rangle {}_{x_{2}},\\ p_{2} & =\text{exp}\left(r\right)|0\rangle {}_{p_{2}}. \end{array}\right.$$ (12)

where *r* ( > 0) is the compression coefficient, and the vacuum state ∣0〉 with the subscript, as the input state, denotes the corresponding quadrature position and quadrature momentum. Then, the 2-mode squeezed states *S*_1_ (signal light) and *S*_2_ (detection light) are generated by these 2 squeezed vacuum states through a 50:50 beam splitter and can be described separately as:

$$\left\{\begin{array}{ll} X_{1} & =\frac{1}{\sqrt{2}}\left(x_{1}+x_{2}\right),\\ P_{1} & =\frac{1}{\sqrt{2}}\left(p_{1}+p_{2}\right). \end{array}\right.$$ (13)

and

$$\left\{\begin{array}{ll} X_{2} & =\frac{1}{\sqrt{2}}\left(x_{2}-x_{1}\right),\\ P_{2} & =\frac{1}{\sqrt{2}}\left(p_{2}-p_{1}\right). \end{array}\right.$$ (14)

Then, *S*^1^, employed as a modulated optical carrier and security detection, is retained by Alice, while ***S2***, employed as a reference for joint measurements and security detection, is sent to Bob.

2. **Security detection**: Alice randomly selects a set of time slots to measure the quadrature components *X* or *P* of *S*_1_ and then she publishes the time slots and the measurements. Bob chooses the same time slots as Alice to measure the corresponding components. Then, the inseparability criterion is relied by Bob to combine the measurements and Alice’s measurements for entanglement judgments, which can be described as follows:

$$\langle {\left(\Delta X_{m}\right)}^{2}\rangle +\langle {\left(\Delta P_{m}\right)}^{2}\rangle <2,$$ (15)

where *X_m_* and *P_m_* represent the measurement results of the corresponding component. The “2” means the sum of the EPR noise that can be observed when each EPR pair is detected separately. If the inseparability criterion is met for the measurement results for all the selected time slot, the information for the location of the time slot is secure. Moreover, the security of the channel is guaranteed when the combined error rate of the measurement results for all selected time slots is below the threshold. Then, the next steps continue; otherwise, the communication is terminated.

3. **Authentication**: Bob publishes measurement results corresponding to the time slot to verify that both parties are legitimate communicating parties for choosing whether to proceed with the next steps, since the classical channel can only be used by legitimate communicators to publish information.
4. **Gaussian mapping**: First, the secret message sequence is divided into multiple information blocks by Alice. Second, the probability of 0 and 1 occurring in each information block is unified using a code conversion method to maintain a mean of 0 for the sequences, thereby reducing the effect on modulation variance. Specifically, 0 in the information block is represented alternately by {01, 10} , while 1 is alternately represented by 00, 11. Third, the information blocks are further divided, and each dividing block is uniformized by randomly replacing all bits with 2-bit code element. Specifically, one of {00, 01} is randomly selected to represent 0, and one of {10, 11} is randomly selected to represent 1. Following that, detection bits are inserted in random positions within each information block. Then, an error correction code is inserted in a fixed position of each information block. Afterward, a Gaussian random sequence *G*_1_ with mean zero and variance *V_A_* is divided into multiple intervals according to the equiprobable rule, where the number of intervals is determined by the number of bits in each information block. Finally, information blocks are mapped to arbitrary Gaussian random numbers that belong to corresponding intervals, resulting in a mapped Gaussian random sequence *G*^1′^(⊆*G*_1_).
5. **Gaussian modulation**: The sequence *G*^′^_1_ is adopted to modulate the quadrature position *X* of *S*_1_, while another Gaussian random sequence *G*_2_(≠*G*^1^^'^) is adopted to modulate the quadrature momentum *P* of *S*_1_. After modulation, *S*_1_ evolves into *S*^info^, which is then transmitted through quantum channel to Bob. The similar modulation scheme [63] is actually based on the one-time pad, in which the *X* component is regarded as the message needed to be transmitted to Bob, while *P* acts as the private key shared between Alice and Bob. Compare to it, in the proposed scheme, one component is used for direct information transmission without encryption, while the other component is solely utilized for parameter estimation.
6. **Measurement**: Joint detection is performed by Bob on detection light *S*^′^_2_(after channel transmission) and signal light *S*^′^_*info*_ for obtaining the corresponding quadrature components, which can be expressed as:

$$\left\{\begin{array}{ll} X_{m}\; & =\frac{1}{\sqrt{2}}\left(X_{\text{info}}^{\prime }-X_{2}^{\prime }\right),\\ P_{m}\; & =\frac{1}{\sqrt{2}}\left(P_{\text{info}}^{\prime }-P_{2}^{\prime }\right). \end{array}\right.$$ (16)

7. **Postprocessing**: The secret message on the basis of obtaining quadrature components is extracted by Alice and Bob through parameter estimation, reverse reconciliation, and message extraction.

### Grading processing method based on parameter estimation

In this section, we introduce the grading processing scheme based on parameter estimation proposed in response to channel fluctuations. Secure message is less difficult to extract from the high-quality information blocks than from the low-quality one, and the high-quality one experiences a better channel environment with less interference. Existing reconciliation strategies can be used effectively to reconciliate for high-quality information blocks, and guarantee low grading process costs and low error rates. On the contrary, the low-quality information blocks suffered by the channel interference and eavesdropping interference more seriously, which means that using the universal reconciliation schemes for processing different quality information blocks requires a great cost and a lot of error correction information to enhance the reconciliation efficiency of low-quality information blocks and to ensure the security of message extraction. Therefore, for the information blocks marked as high quality or low quality, choosing a matching reconciliation strategy is beneficial to improve the reconciliation efficiency of information blocks and speed up the extraction rate of secret message.

•**Scheme structure**

**Fig. 7.**
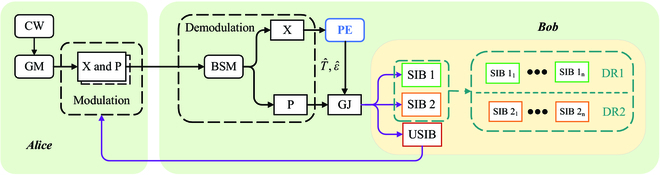
CV-QSDC parameter estimation scheme. GM, Gaussian modulation; GJ, grading judgement; BSM, Bell-state measurement; SIB1, high-level information blocks; SIB2, low-level information blocks; USIB, unsafe information block; PE, parameter estimation; DR, different reconciliation schemes applying to different quality information blocks.

Figure 7 details the CV-QSDC parameter estimation scheme, which mainly includes secure judgment and grading processing process. The basic steps of the scheme are as follows:

**Step 1**: Bell-state measurement: Bob employs Bell-state measurement to measure the information blocks from Alice for obtaining 2 sets of variables modulated onto the *X* and *P* components.

**Step 2**: Parameter estimation: The set of variables *X* that do not carry secret message is selected as the sample of parameter estimation, and 2 judgment criteria are set as grading criteria based on the results of parameter estimation.

**Step 3**: Security detection: Since the fiber channel is relatively stable, the estimation of secrecy capacity of *i*-th information blocks $\Delta {\hat{I}}_{i}$ should fluctuate within a relatively narrow band, and there must exist eavesdropping behavior if this estimate is not normal or below zero. Therefore, a relevant criterion is employed for eavesdropping detection.

**Step 4**: Quality judgment: The mean of secrecy capacity $\Delta {\bar{I}}_{i}$ can be used as a measure of signal classification and provides a reliable basis for different reconciliation strategies in the grading processing phase. Therefore, the first criterion is employed to judge the safety of information blocks, and the other criterion is employed to judge the quality of information blocks on the basis of the previous criterion.

•**Criteria for quality judgment**

Generally, the secrecy capacity Δ*I* between Alice and Bob is given by [64]

$$\Delta I=\left(1-\mathrm{FER}\right)\left(1-P\right)\left(\beta I_{\mathrm{AB}}-\chi_{\mathrm{BE}}\right),$$ (17)

where *β* is the efficiency of grading reconciliation algorithm, *I*_AB_ is the mutual information between Alice and Bob, *χ*_BE_ is the Holevo bound between Eve and Bob, and *P* is the error rate of information block. *FER* is the frame error rate of the reconciliation, which can be used to evaluate the reliability and efficiency of the reconciliation scheme. The estimation of secrecy capacity $\Delta {\hat{I}}_{i}=\left\{\Delta {\hat{I}}_{1},\Delta {\hat{I}}_{2},\ldots ,\Delta {\hat{I}}_{M}\right\}$ of the *i*-th information block (*i* ∈ {1, 2, …, *M*}) can be calculated through the estimation of channel transmittance ${\hat{T}}_{i}=\left\{{\hat{T}}_{1},{\hat{T}}_{2},\ldots ,{\hat{T}}_{i}\right\}$ and excess noise ${\hat{\varepsilon }}_{i}=\left\{{\hat{\varepsilon }}_{1},{\hat{\varepsilon }}_{2},\ldots ,{\hat{\varepsilon }}_{i}\right\}$ from Eqs. 5 to 10.

The parameters involved in the grading criterion calculation process are all obtained based on the *X* component of information block and the pilot signals that are designed with reference to the synchronization frame [54]. The pilot signals consist of quantum light undergoing the same channel as the transmitted signal light. Therefore, the prior information such as reconciliation efficiency obtained from these pilot signals and the *X* component can be used for synchronization and also for calculating the average secrecy capacity of the blocks and grading the information blocks. After grading the information blocks, the *P* component of information blocks undergoes different reconciliation processes according to the grading results.

**Criterion 1**: Determines the security of *i*-th information block based on whether the secrecy capacity $\Delta {\hat{I}}_{i}$ is greater than zero or not,

$$\left\{\begin{array}{cc} \Delta {\hat{I}}_{i}\; & >0,\;\text{safe block},\\ \Delta {\hat{I}}_{i}\; & \leq 0,\;\text{unsafe block}. \end{array}\right.$$ (18)

**Criterion 2**: In the *i*-th information block, $\Delta {\hat{I}}_{i}$ is employed as a basis for judging transmission performance. By comparing it with the average of the previous secrecy capacity $\Delta \bar{I}=\frac{1}{i}\sum_{k=1}^{i}‍\Delta {\hat{I}}_{k}$, the quality of the current information block in broad terms can be divided into high level and low level

$$\left\{\begin{array}{cc} \Delta {\hat{I}}_{i}\; & \geq \Delta \bar{I},\;\text{high level},\\ \Delta \bar{I}\; & >\Delta {\hat{I}}_{i},\;\text{low level}. \end{array}\right.$$ (19)

In the 2 criteria mentioned above, the secrecy capacity is compared with zero in criterion 1***,*** which is the criterion for appraising whether the information block is interfered by eavesdropping during transmission. The security capacity is compared with the mean value of the security capacity $\Delta \bar{I}.$ in criterion 2, which is used to evaluate whether the information block is of low or high quality. The mean value of the secrecy capacity is actually obtained by calculating the secrecy capacity for *X* component of multiple blocks and then obtaining the $\Delta \bar{I}$.

Under the combined effect of the 2 criteria, the information blocks are divided into 3 types: SIB1, SIB2, and USIB. Both SIB1 and SIB2 meet both 2 criteria. The former is classified as high-level information blocks, while the latter is classified as low-level information blocks. However, the unsafe information blocks that do not meet criterion 1 are represented by USIB and do not perform subsequent information processing.

Based on the above grading criteria, the grading process is designed as follows: After receiving the *i*-th information block from Alice, Bob first calculates the estimate of the relevant parameter by Eqs. 5 to 10 for obtaining the secrecy capacity $\Delta {\hat{I}}_{i}$ of *i*-th information block (*i* ∈ {1, 2, …, *M*}) by Eq. 17. Then, the secrecy capacity $\Delta {\hat{I}}_{i}$ is employed for determining the security of the block according to criterion 1. If it is judged to be unsafe, it would be retransmitted until it is judged to be safe. But if it is judged to be secure, Bob would further determine the quality of the information block according to criterion 2. According to the judgment result, the information block is classified as a high-level block or a low-level block, and then we proceed to the next information block. As shown in Fig. 7, DR1 represents a reconciliation scheme applied to high-level information, while DR2 represents a reconciliation scheme applied to low-level information. Since the reconciliation performance is affected by the transmission environment such as the signal-to-noise ratio. According to the classification of transmission quality in this scheme, different reconciliation strategies can be designed for the information blocks of different transmission quality to improve the grading processing performance and lay the groundwork for the grading processing reconciliation scheme. It conduces to maintain the invariant and efficient reconciliation efficiency under fluctuating channel and facilitates the subsequent complete message extraction.

•**Validation**

The calculation result of the estimated secrecy capacity may effectively reflect the transmission quality of the information blocks. Estimates of secrecy capacity for all information blocks are shown in Fig. 6. According to the criteria for quality judgment, criterion 1 for determining the security of an information block is whether the secrecy capacity is greater than zero, while criterion 2 for grading the information block as high level or low level is whether the secrecy capacity is greater than the average secrecy capacity. The experimental results show that all information blocks meet criterion 1, that is, security message in each information block exists, indicating that the communication process is secure. Most of the yellow data points that are judged as high-level blocks according to criterion 2 are located above the green data points, which are judged as low-level blocks according to criterion 2, indicating that the information block is basically divided into 2 categories of high level and low level. Moreover, dividing the information blocks that the secrecy capacity is around the average into data blocks with high or low level has little impact on subsequent data reconciliation, so the impact of grading processing for them can be ignored. These results demonstrate the security of the experimental communication process and the feasibility and effectiveness of the grading processing scheme.

Based on the above analysis, the feasibility of the grading processing scheme based on secrecy capacity is explained. Moreover, according to the analysis in the “Parameter estimation scheme” section, the secrecy capacity in information blocks is determined by the channel transmittance and excess noise. The estimation of the channel transmittance and excess noise is shown to be effective in Fig. 4. Therefore, the estimation of secrecy capacity and the grading criteria of information in this protocol are also effective. The transmission length of the fiber channel is set to 5 km in the experimental scheme. Therefore, the theoretical value of the secrecy capacity of the information block can be calculated by Eq. 17 and represented by a blue dotted line in Fig. 6. Furthermore, all data points are below the blue dotted line, indicating that the optimization of the experimental system is constantly approaching the theoretical analysis. Moreover, the comparison of the performance impact of quality grading on information blocks is shown in Fig. 5 according to experiments and analysis. The performance of the system that does not implement grading is represented by blue lines, while the performance of the system that has implemented grading is represented by yellow lines, which indicates the improvement of classification on system performance.

## Data Availability

Data sharing is not applicable to the article as no datasets were generated or analyzed during the current study.
